# Role of SATB2 in human pancreatic cancer: Implications in transformation and a promising biomarker

**DOI:** 10.18632/oncotarget.10860

**Published:** 2016-07-27

**Authors:** Wei Yu, Yiming Ma, Sharmila Shankar, Rakesh K. Srivastava

**Affiliations:** ^1^ Kansas City VA Medical Center, Kansas City, MO 66128, USA; ^2^ Department of Pathology, University of Missouri-School of Medicine, Kansas City, MO 64108, USA; ^3^ Department of Pharmaceutical Sciences, University of Missouri-School of Medicine, Kansas City, MO 64108, USA

**Keywords:** pancreatic cancer, SATB2, cancer stem cell, stem cell, pluripotency

## Abstract

SATB2 (special AT-rich binding protein-2), a transcription factor and chromatin modulator, regulates the expression of genes required for maintaining pluripotency and self-renewal. The molecular mechanisms by which human pancreatic normal ductal epithelial cells are transformed to cancer cells are not well understood. The main goal of the paper is to examine the molecular mechanisms by which SATB2 regulates transformation of human pancreatic normal ductal epithelial (HPNE) cells, and assess whether transformed HPNE cells gained the phenotypes of cancer stem cells (CSCs). The results demonstrate that SATB2 is highly expressed in pancreatic CSCs, primary tissues and cell lines, but not in HPNE cells. SATB2 induces cellular transformation, stemness and epithelial to mesenchymal transition in HPNE cells, and inhibition of its expression suppresses these activities. Overexpression of SATB2 in HPNE cells resulted in induction of stem cell markers (CD44, CD24 and CD133), and transcription factors (Oct4, Sox2 and Nanog). SATB2 can directly bind to promoters of Bcl-2, Bsp, Nanog, c-Myc, XIAP, Klf4 and Hoxa2, suggesting the role of SATB2 in pluripotency, cell survival and proliferation. SATB2-overexpressing HPNE cells (HPNE/SATB2) formed tumors in Balb C nude mice, whereas HPNE/Empty vector cells did not form any tumor. Since SATB2 is highly expressed in human pancreatic cancer tissues and cell lines, but not in HPNE cells and normal pancreatic tissue, it can drive pancreatic cancer growth and metastasis. Our findings suggest that SATB2 can induce dedifferentiation by inducing stemness and may have a role in pancreatic carcinogenesis, and can be used as a diagnostic biomarker.

## INTRODUCTION

Pancreatic cancer is one of the leading causes of cancer-related deaths in the Western world, and its incidences are increasing [1]. It is the most deadly disease with a 5-year survival rate of less than 6% [1]. Some of the characteristics of the pancreatic cancer include poor prognosis, late discovery due to silent growth, and resistance to chemotherapy and radiation [2]. Unfortunately, at the time of diagnosis many pancreatic cancers are not resectable due to metastasis to the regional lymph nodes and distant organs, and these characteristics make the management of pancreatic cancer very difficult [3]. Several factors such as genetic, environmental carcinogen, diet and lifestyle may cause pancreatic cancer [4]. Recent studies have demonstrated the contribution of cancer stem cells (CSCs)/tumor initiating cells in tumor initiation, promotion, and metastasis [2]. Our recent studies showed the existence of CSCs in pancreatic tissues isolated from human and Kras^G12D^ mice [5–8]. These cells were similar to normal stem cells and expressed cell surface markers such as CD24, CD44, ESA and CD133 [2]. Although pancreatic CSCs isolated from human primary tumors and Kras^G12D^ mice are tumorigenic, the molecular mechanisms by which human pancreatic normal ductal epithelial (HPNE) cells are transformed to malignant phenotype are not well understood.

SATB2 (special AT-rich binding protein-2), a transcription factor and epigenetic regulator [9], regulates gene expression both by modulating chromatin architecture and by functioning as a transcriptional factor [10–14]. The SATB2 gene is conserved in humans and mouse. In humans, there are three transcripts which encodes for SATB2 protein. Human and mouse share three Oct-4, one Nanog and two c-Myc binding sites on chromosome 2. *SATB2*^−/−^ mice are defective in bone development and osteoblast differentiation [11]. It is linked to craniofacial patterning and osteoblast differentiation [11], and in development of cortical neurons [12–15]. SATB2 is over expressed in 85% of CRC tumors, suggesting its use as a diagnostic marker for colon cancer [16]. In breast cancer, SATB2 mRNA expression is significantly associated with cancer progression and poor survival [17]. However, the tumor promoting and metastatic roles SATB2 in pancreatic carcinogenesis have never been examined. Stem cells heavily depend on the pluripotency maintaining factors (Nanog, Oct-4, Sox-2 and Klf-4) for their self-renewal and survival. We have demonstrated that pancreatic CSCs expressing CD24, CD44, CD133 and ESA are highly tumorigenic in NOD/SCID/IL2Rϒ^null^ mice [7]. Since SATB2 binding sites are present on Oct-4, Nanog and c-Myc, it can directly regulate their expressions and stem cell characteristics. Furthermore, the modulation of SATB2 in HPNE cells may have a significant role in cellular transformation and pancreatic carcinogenesis because majority of pancreatic adenocarcinomas are developed from HPNE cells.

The objective of the paper was to assess whether SATB2 is capable of inducing malignant transformation in human pancreatic normal ductal epithelial cells, and these transformed cells gained the phenotype of CSCs and are capable of forming tumors in nude mice. We have identified, for the first time, that SATB2 is highly expressed in human pancreatic CSCs (Pan CSCs) and cancer cell lines, but not in human pancreatic normal ductal epithelial cells. Using ChIP assay, we have demonstrated that SATB2 can directly bind to the promoters of Nanog, c-Myc, Klf-4, Bcl-2, Bsp, XIAP, and Hoxa2 genes, suggesting SATB2 can act as a master regulator of pluripotency and self-renewal in pancreatic CSCs. Overexpression of SATB2 in HPNE cells induces malignant transformation, whereas inhibition of SATB2 expression in Pan CSCs suppresses cell growth, motility and colony formation. SATB2-transformed HPNE cells gained the phenotypes of CSCs and also formed tumors in nude mice, whereas HPNE/empty vector group did not form any tumor in mice. The overexpression of SATB2 in Pan CSC tumors was positively co-related with the expression of Oct-4, Nanog, c-Myc and Sox-2. These studies will enhance our understanding of the role of SATB2 in malignant transformation, tumor growth and metastasis.

## RESULTS

### SATB2 is highly expressed in pancreatic cancer stem cells (CSCs) and cell lines, but not in human pancreatic normal ductal epithelial (HPNE) cells

**Figure 1:**
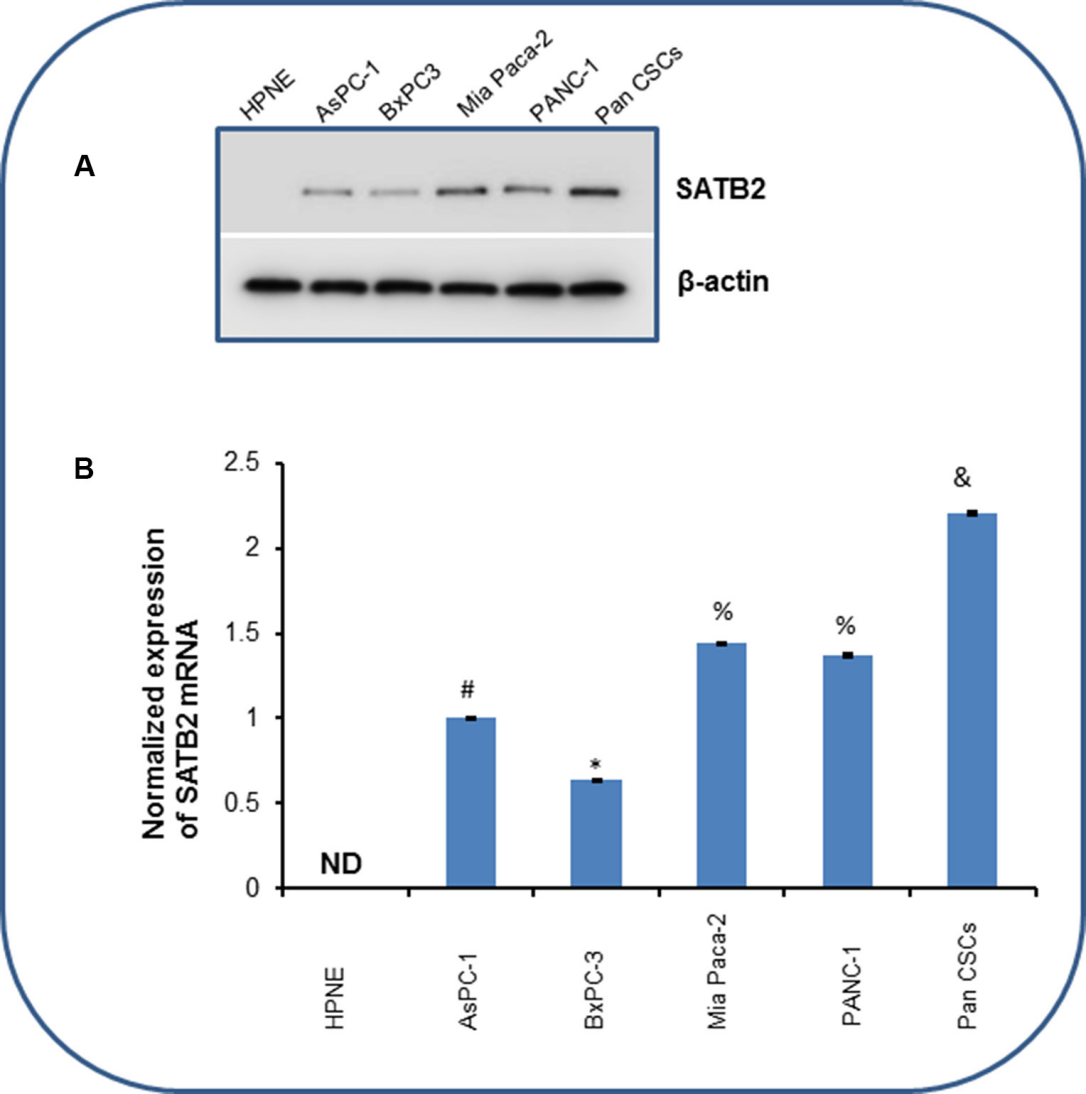
The expression of SATB2 in HPNE, pancreatic cancer cell lines and pancreatic CSCs (**A**) Protein expression of SATB2 in human pancreatic normal ductal epithelial cells (HPNE), pancreatic cancer cell lines and Pan CSCs. Crude proteins were isolated and the expression of SATB2 was measured by the Western blot analysis. β-actin was used as a loading control. (**B**) Expression of SATB2 mRNA in HPNE, pancreatic cancer cell lines and Pan CSCs. RNA was isolated and the expression of SATB2 was measured by qRT-PCR. GAPDH was used as an internal control. Data represent mean (*n* = 4) ± SD. *, #, & and % = significantly different from HPNE (*P* < 0.05). ND = Not Detected. Pan CSCs = Pancreatic Cancer Stem Cells.

SATB2 is a transcription factor which plays a significant role in self-renewal and pluripotency [11]. In order to understand the biological function of SATB2, we first compared its expression in HPNE cells, pancreatic cancer cell lines (AsPC-1, BxPC-3, Mia-PaCa-2, and PANC-1) and Pan CSCs by Western blot analysis and qRT-PCR. SATB2 is not expressed in HPNE cells. However, it is highly expressed in pancreatic cancer cell lines and Pan CSCs (Figure 1). Interestingly, the expression of SATB2 was highest in Pan CSCs compared to that of AsPC-1, BxPC-3, Mia-PaCa-2, and PANC-1 cells. These data suggest that the expression of SATB2 is tightly regulated in pancreatic cancer/transformed cells, and it may have a role in malignant transformation.

### Overexpression of SATB2 in HPNE cells induces cellular transformation and stemness (by expressing stem cell markers and pluripotency maintaining factors)

**Figure 2:**
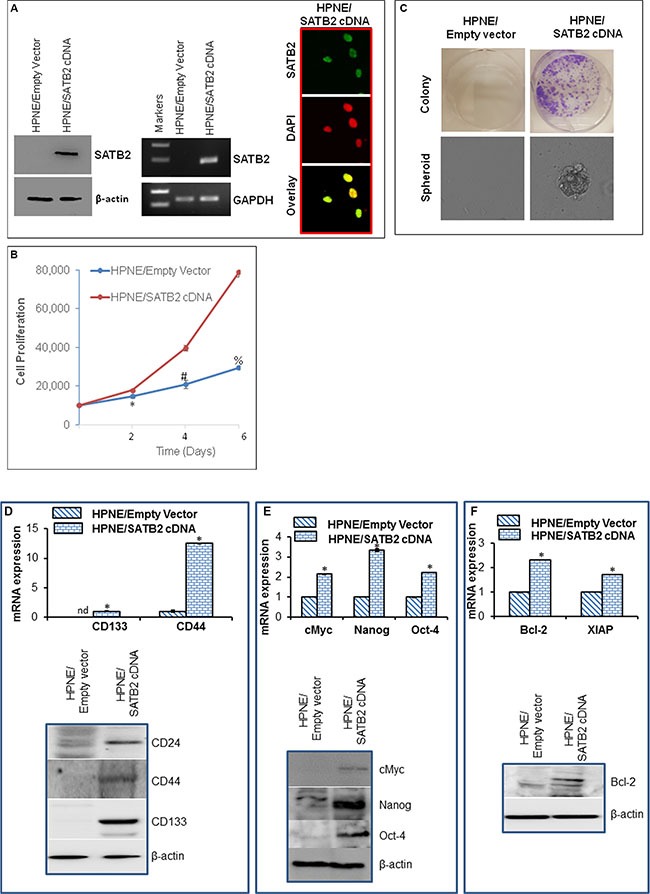
Overexpression of SATB2 in HPNE cells induces cellular transformation and stemness (**A**) HPNE cells were stably transduced with lentiviral particles expressing either empty vector or SATB2 cDNA. SATB2 expression was measured by the Western blot analysis, RT-PCR and immunocytochemistry. (**B**), Proliferation of HPNE/Empty Vector and HPNE/SATB2 cDNA cells was measured for 6 days. Data represent mean (*n* = 4) ± SD. *, # and % = significantly different from respective empty vector groups, *P* < 0.05. (**C**), Colony and spheroid formation. Colony formation in soft agar and spheroid formation in suspension of HPNE/Empty Vector and HPNE/SATB2 cDNA cells were measured. (**D**) Upper panel, RNA expression of stem cell markers. RNA was isolated and the expression of stem cell markers (CD133 and CD44) was measured by qRT-PCR analysis. Data represent mean (*n* = 4) ± SD. * = significantly different from HPNE/Empty Vector group (*P* < 0.05). Gene expression of HPNE/Empty Vector cells was normalized to 1. Lower panel, Protein expression of stem cell markers. Cell lysates were collected from HPNE/Empty Vector and HPNE/SATB2 cDNA cells, and the expression of CD24, CD44 and CD133 was measured by the Western blot analysis. β-actin was used as a loading control. (**E**) Upper panel, RNA was isolated and the expression of transcription factors (c-Myc, Nanog and Oct-4) was measured by qRT-PCR analysis. Data represent mean (*n* = 4) ± SD. * = significantly different from HPNE/Empty Vector group (*P* < 0.05). Gene expression of HPNE/Empty Vector cells was normalized to 1. Lower panel, Protein expression of c-Myc, Nanog and Oct-4 Cell lysates were collected from HPNE/Empty Vector and HPNE/SATB2 cDNA cells, and the expression of c-Myc, Nanog and Oct-4 was measured by the Western blot analysis. β-actin was used as a loading control. (**F**), Upper panel, RNA was isolated and the expression of Bcl-2 and XIAP was measured by qRT-PCR analysis. Data represent mean (*n* = 4) ± SD. * = significantly different from HPNE/Empty Vector group (*P* < 0.05). Gene expression of HPNE/Empty Vector cells was normalized to 1. Lower panel, Protein expression of Bcl-2. Cell lysates were collected from HPNE/Empty Vector and HPNE/SATB2 cDNA cells, and the expression of Bcl-2 was measured by the Western blot analysis. β-actin was used as a loading control.

The cell transformation characteristics are high/indefinite saturation density, no contact inhibition, less oriented growth, loss of tight junction and the formation of colonies. In order to prove that SATB2 induces cellular transformation and stemness, we overexpressed SATB2 in HPNE wild type cells. Lentiviral-mediated infection of SATB2 gene in HPNE (HPNE/SATB2) cells resulted in an increased expression of SATB2 protein and mRNA, as tested by the Western blotting, RT-PCR and immunocytochemistry (Figure 2A). Furthermore, HPNE/SATB2 cells demonstrated enhanced cell growth compared to HPNE/empty vector cells (Figure 2B).

We next examined whether SATB2 induces transformation, and transformed cells gained stemness by expressing stem cell markers and pluripotency maintaining factor. Overexpression of SATB2 gene induced cellular transformation as evident by formation of colonies and spheroids in suspension (Figure 2C). Normal HPNE cells (HPNE/empty vector) were unable to form colonies in soft agar and spheroids in suspension. Overall, these data suggest that overexpression of SATB2 gene is capable of inducting stem cell phenotype.

Since SATB2 transformed cells exhibited enhanced cell proliferation and formed colonies and spheroids, we next sought to examine the effects of SATB2 on the expression of stem cell markers, pluripotency maintaining factors and cell survival proteins. Overexpression of SATB2 in HPNE cells resulted in induction of stem cell markers (CD24, CD44, and CD133), and transcription factors (c-Myc, Nanog and Oct-4) and upregulation of cell survival Bcl-2 and apoptosis inhibitor XIAP (Figure 2D–2F). Overall, these data suggest that SATB2 can induce cellular transformation in HPNE cells by inducing stemness and regulating cell survival/proliferation.

### SATB2 directly binds to Bcl-2, Bsp, Nanog, c-Myc, XIAP, Klf4 and Hoxa2 in Pan CSCs

**Figure 3:**
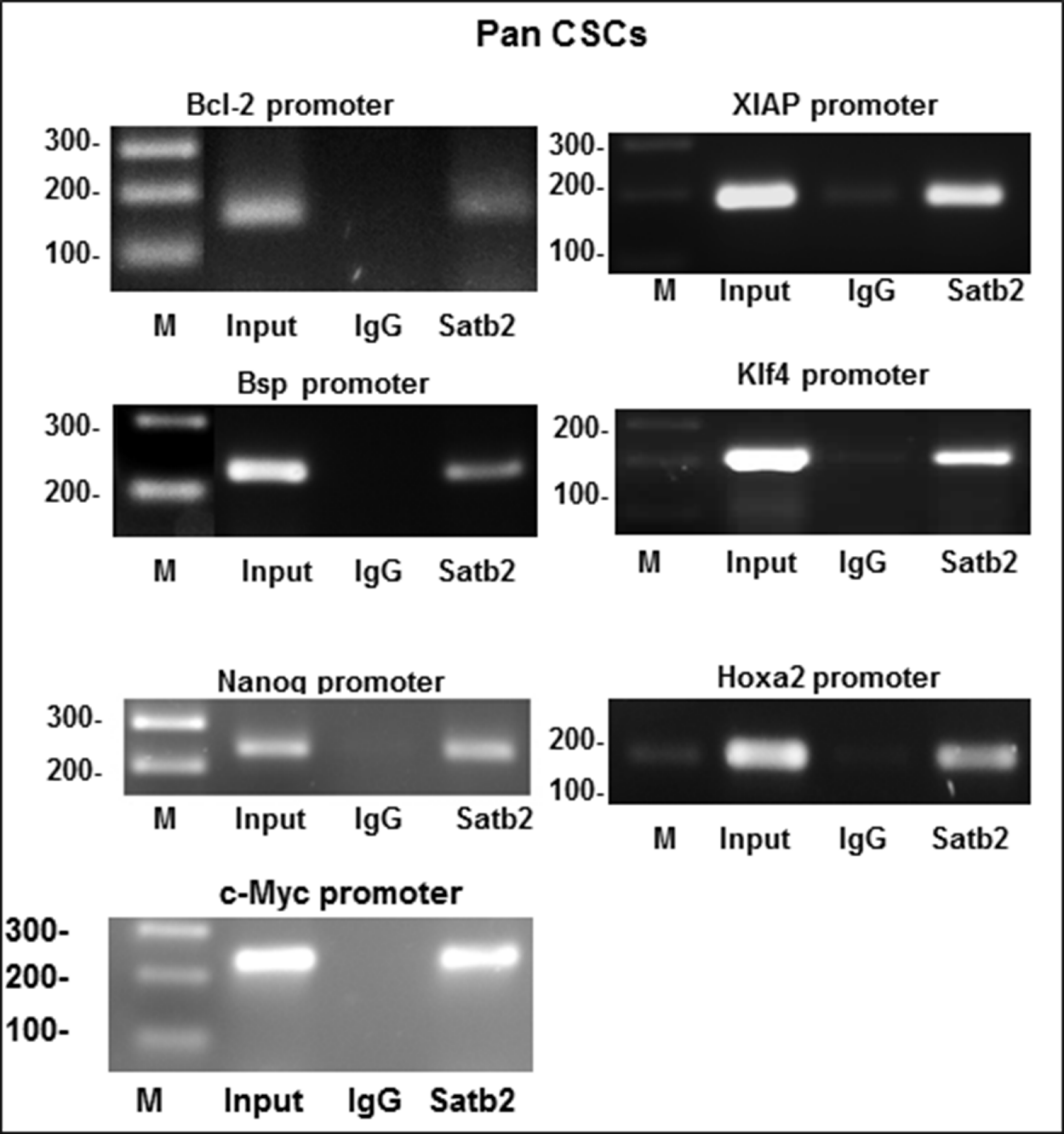
Binding of SATB2 to promoters of Bcl-2, Bsp, Nanog, c-Myc, XIAP, Klf4 and Hoxa2 Nuclear extracts were prepared from pancreatic CSCs. Chromatin immunoprecipitation (ChIP) assays were performed to examine the binding of the SATB2 to the promoters of Bcl-2, Bsp, Nanog, c-Myc, XIAP, Klf4 and Hoxa2 in pancreatic CSCs.

SATB2 is a transcription factor which regulates stemness, cell proliferation and survival [18]. We therefore examined whether SATB2 can directly bind to Bcl-2, Bsp, Nanog, c-Myc, XIAP, Klf4 and Hoxa2 which regulate stemness, cell growth and survival. Chromatin immunoprecipitation (ChIP) assays were performed to examine the binding partners of SATB2 (Figure 3). SATB2 can directly bind to promoters of Bcl-2, Bsp, Nanog, c-Myc, XIAP, Klf4 and Hoxa2 as demonstrated by ChIP assay. These data suggest that SATB2 can regulate several cellular functions by regulating pluripotency, cell survival and proliferation genes.

### Overexpression of SATB2 in HPNE cells induces epithelial to mesenchymal transition

**Figure 4:**
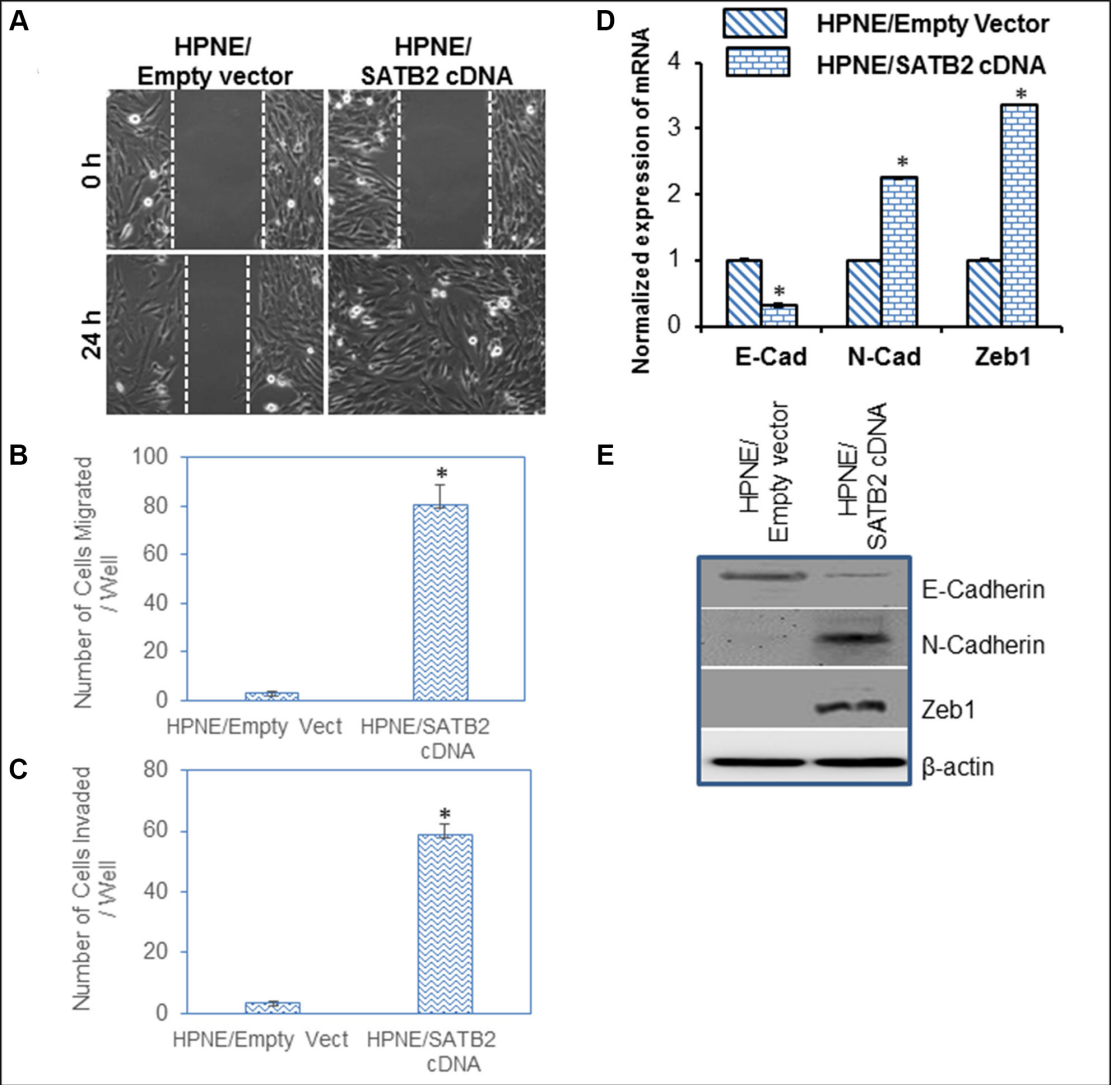
Overexpression of SATB2 in HPNE cells induces EMT characteristics (**A**) Cell Motility assay. HPNE/Empty Vector and HPNE/SATB2 cDNA cells were grown in petri dishes. After cell attachment, scratch lines were made with the fine pipette tips in both the groups. Phase contrast images of cells were captured at 0 h and 24 h time points. (**B**) Transwell Migration Assay. 1 × 10^5^ cells were plated on to the top chamber of the noncoated membrane (24-well insert; pore size, 8 μm; Corning Costar) and allowed to migrate toward serum-containing medium in the lower chamber. Cells were fixed after 24 hours of incubation with methanol and stained with Diff-Quick Fixative Solutions. Data represent mean (*n* = 4) ± SD. * = significantly different at *P* < 0.05. (**C**) Transwell Invasion Assay. 1 × 105 cells were plated on to the top chamber of the Matrigel coated Membrane. After 48 hours, Matrigel-coated inserts were fixed and stained. The number of cells invading through the membrane was counted. Data represent mean ± SD. * = significantly different at *P* < 0.05. (**D**) Expression of E-Cadherin, N-cadherin and Zeb1 in HPNE/Empty Vector and HPNE/SATB2 cDNA cells. RNA was isolated and the expression of E-Cadherin, N-cadherin and Zeb1 was measured by qRT-PCR analysis. Data represent mean (*n* = 4) ± SD. * = significantly different from HPNE/Empty Vector group (*P* < 0.05). Gene expression of HPNE/Empty Vector group was normalized to 1. (**E**), Protein expression of E-Cadherin, N-cadherin and Zeb1. Cell lysates were collected from HPNE/Empty Vector and HPNE/SATB2 cDNA cells, and the expression of E-Cadherin, N-cadherin and Zeb1 was measured by the Western blot analysis. β-actin was used as a loading control.

Epithelial to mesenchymal transition (EMT) is a process whereby epithelial cells undergo transition to a mesenchymal phenotype and contribute directly to stemness and cancer cell metastasis [19]. We therefore sought to examine whether overexpression of SATB2 in normal HPNE cell enhances cell motility, migration and invasion. As shown in Figure 4A–4C, overexpression of SATB2 in HPNE cells enhanced cell motility, migration and invasion. The transcription factor Zeb1 regulate the expression of cadherins during EMT. We therefore compared the expression of E-cadherin, N-cadherin and Zeb1 in HPNE/Empty Vector and HPNE/SATB2 cDNA cells. Overexpression of SATB2 resulted in induction of Zeb1 and N-cadherin, and inhibition of E-cadherin at both mRNA and protein levels in HPNE/SATB2 cDNA cells compared to HPNE/Empty Vector cells (Figure 4D and 4E). These data suggest that SATB2 gene is capable of inducing EMT in HPNE cells.

### Knockdown of SATB2 in Pan CSCs and pancreatic cancer cell lines inhibits cell proliferation, and colony formation

**Figure 5:**
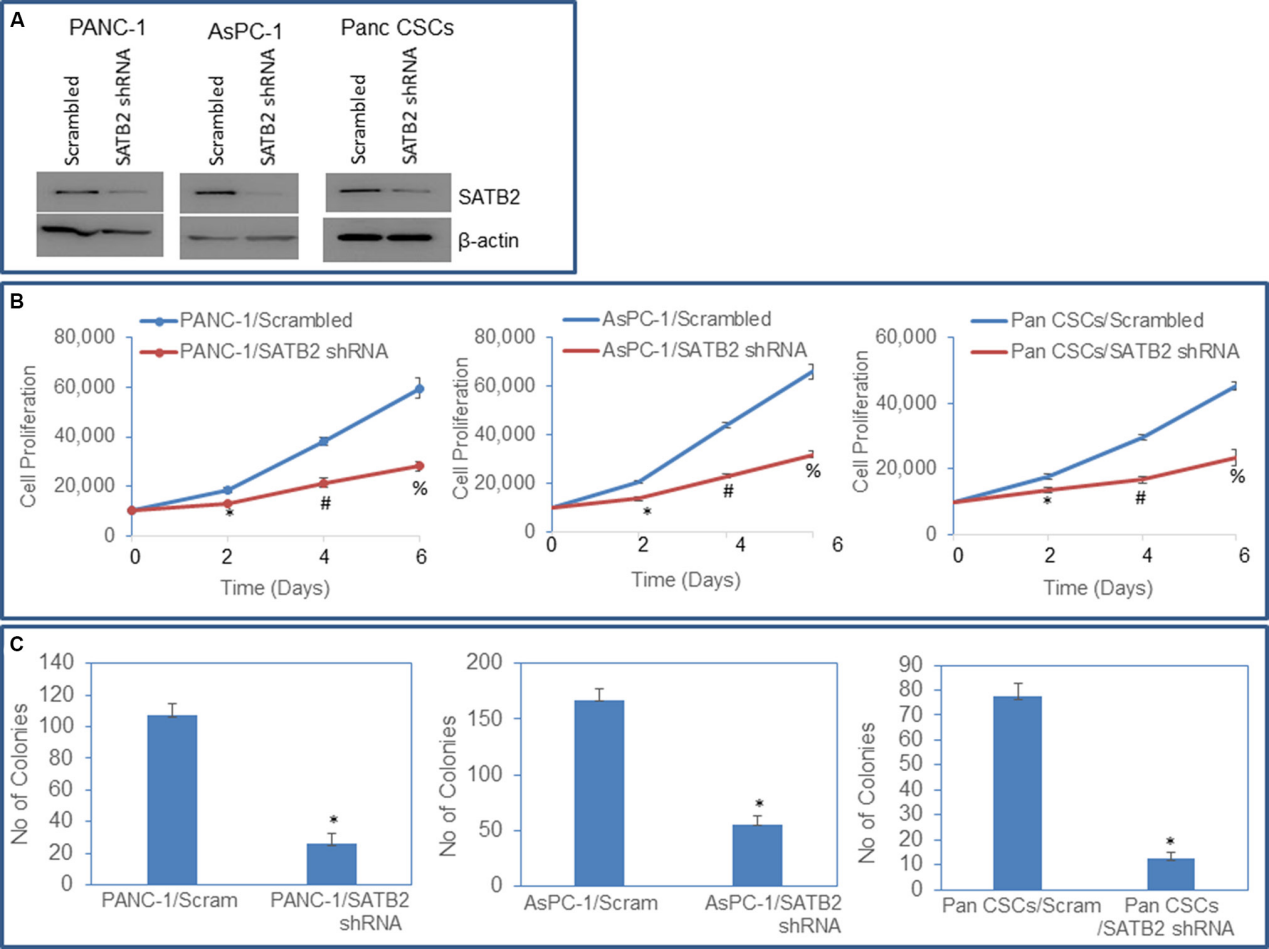
SATB2 shRNA inhibits cell proliferation and colony formation in pancreatic cancer cells and Pan CSCs (**A**) Expression of SATB2. PANC-1, AsPC-1 and Pan CSCs were transduced with either scrambled or SATB2 shRNA. Cell lysates were collected and the expression of SATB2 was measured by the Western blot analysis. β-actin was used as a loading control. (**B**) Cell proliferation of PANC-1/Scrambled, PANC-1/SATB2 shRNA, AsPC-1/Scrambled, AsPC-1/SATB2 shRNA, Pan CSCs/Scrambled and Pan CSCs/SATB2 shRNA groups was measured over 6-day period. (**C**) Colony formation Assay. PANC-1/Scrambled, PANC-1/SATB2 shRNA, AsPC-1/Scrambled, AsPC-1/SATB2 shRNA, Pan CSCs/Scrambled and Pan CSCs/SATB2 shRNA cells were seeded, and number of colonies formed at 21 days were counted. Data represent mean (*n* = 4) ± SD. * = significantly different from HPNE empty vector (*P* < 0.05).

SATB2 plays an important role in the chromatin remodeling and regulation of gene expression which participates in stemness, cell survival and differentiation [11, 13, 20, 21]. We next examined whether inhibition of SATB2 attenuates the growth of pancreatic cancer PANC-1 and AsPC-1 cells and Pan CSCs. PANC-1, AsPC-1 and Pan CSCs were transduced with either scrambled or SATB2 shRNA lentiviral particles, and SATB2 expression and cell growth were measured. Knockdown of SATB2 by shRNA inhibited SATB2 expression as measured by the Western blot analysis (Figure 5A). PANC-1/SATB2 shRNA, AsPC-1/SATB2 shRNA and Pan CSCs/SATB2 shRNA groups had lower growth rates than PANC-1/Scrambled, AsPC-1/Scrambled, and Pan CSCs/Scrambled groups, respectively (Figure 5B). These data suggest that the knockdown of SATB2 in pancreatic cancer cells and CSCs can suppress pancreatic cancer cell proliferation. We next examined the effects of SATB2 on colony formation. The inhibition SATB2 expression by shRNA attenuated colony formation in PANC-1/SATB2 shRNA, AsPC-1/SATB2 shRNA and Pan CSCs/SATB2 shRNA groups compared to their respective scrambled control groups (Figure 5C). Taken together, these data suggest that inhibition of SATB2 expression in pancreatic cancer cells and CSCs can retard cell proliferation and colony formation.

### Knockdown of SATB2 in Pan CSCs and pancreatic cancer cell lines inhibits epithelial-mesenchymal transition, and markers of cell proliferation, pluripotency and stem cells

**Figure 6:**
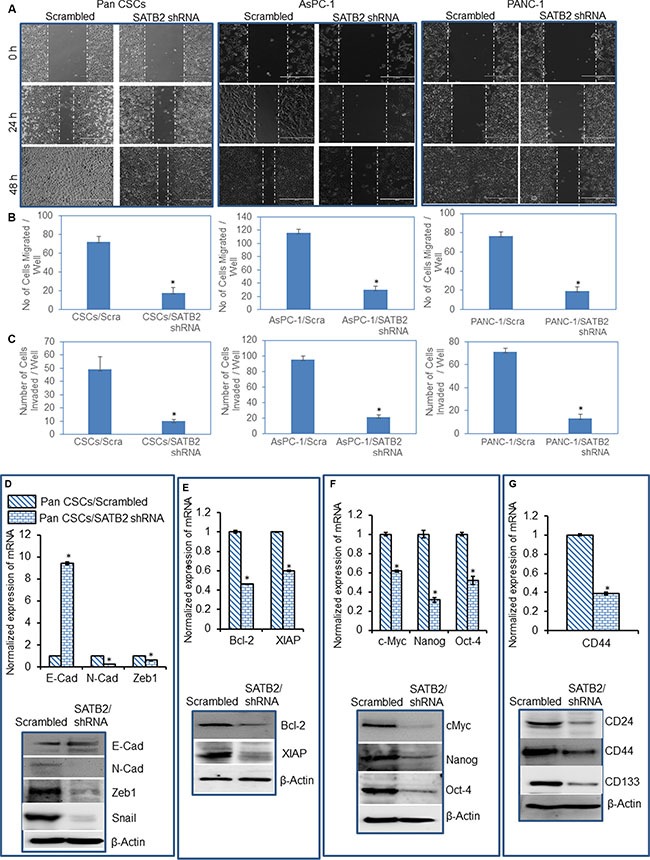
Knockdown of SATB2 in pancreatic cancer cell lines inhibits epithelial-mesenchymal transition, and markers of cell proliferation, pluripotency and stem cells (**A**) Cell Motility Assay. Pan CSCs/Scrambled, Pan CSCs/SATB2 shRNA, AsPC-1/Scrambled, AsPC-1/SATB2 shRNA, PANC-1/Scrambled, PANC-1/SATB2 shRNA cells were grown in petri dishes. After 18 hours of incubation, cells were scratched with the fine pipette tips. Phase contrast images of scratched cells were captured at 0 h, 24 h and 48 h time points. (**B**) Transwell Migration Assay. Transwell migration assay was performed in Pan CSCs/Scrambled, and Pan CSCs/SATB2 shRNA cells as described in Materials and Methods. Data represent mean ± SD. * = significantly different at *P* < 0.05. (**C**) Transwell Invasion Assay. Transwell invasion assay was performed in Pan CSCs/Scrambled and Pan CSCs/SATB2 shRNA as described in Materials and Methods. Data represent mean ± SD. * = significantly different at *P* < 0.05. (**D**) Expression of EMT-related genes/proteins. Upper panel, RNA was isolated and the expression of E-cadherin, N-cadherin, and Zeb1 in Pan CSCs/Scrambled, and Pan CSCs/SATB2 shRNA cells was measured by qRT-PCR. GAPDH was used as an internal control. Data represent mean (*n* = 4) ± SD. * = significantly different between groups (*P* < 0.05). Lower panel, Protein expression of E-Cadherin, N-cadherin, Zeb1 and Snail. Cell lysates were collected, and the expression of E-Cadherin, N-cadherin, Zeb1 and Snail was measured by the Western blot analysis. β-Actin was used as a loading control. (**E**) Expression of cell proliferation/survival genes/proteins. Upper panel, RNA was isolated and the expression of Bcl-2 and XIAP in Pan CSCs/Scrambled, and Pan CSCs/SATB2 shRNA cells was measured by qRT-PCR. GAPDH was used as an internal control. Data represent mean (*n* = 4) ± SD. * = significantly different between groups (*P* < 0.05). Lower panel, Protein expression of Bcl-2 and XIAP. Cell lysates were collected, and the expression of Bcl-2 and XIAP was measured by the Western blot analysis. β-Actin was used as a loading control. (**F**) Expression of pluripotency maintaining factors. Upper panel, RNA was isolated and the expression of c-Myc, Nanog, and Oct-4 in Pan CSCs/Scrambled, and Pan CSCs/SATB2 shRNA cells was measured by qRT-PCR. GAPDH was used as an internal control. Data represent mean (*n* = 4) ± SD. * = significantly different between groups (*P* < 0.05). Lower panel, Protein expression of c-Myc, Nanog, and Oct-4. Cell lysates were collected, and the expression of c-Myc, Nanog, and Oct-4 was measured by the Western blot analysis. β-Actin was used as a loading control. (**G**) Expression of stem cell markers. Upper panel, RNA was isolated and the expression of CD44 in Pan CSCs/Scrambled, and Pan CSCs/SATB2 shRNA cells was measured by qRT-PCR. GAPDH was used as an internal control. Data represent mean (*n* = 4) ± SD. * = significantly different between groups (*P* < 0.05). Lower panel, Protein expression of stem cell markers. Cell lysates were collected, and the expression of CD24, CD44 and CD133 was measured by the Western blot analysis. β-Actin was used as a loading control.

An epithelial to mesenchymal transition (EMT) is a process of cell remodeling and appears to be essential during embryonic development, organogenesis and carcinogenesis [22, 23]. During an EMT, epithelial cells lose their polarized organization and acquire migratory and invasive capabilities [24, 25]. We therefore examined whether inhibition of SATB2 attenuates EMT characteristics in pancreatic cancer cells and CSCs. Pan CSCs, AsPC-1 and PANC-1 cells were transduced with either scrambled or SATB2 shRNA lentiviral particles as described above. SATB2 shRNA inhibited the cell motility, cell migration and invasion in Pan CSCs, AsPC-1 and PANC-1 cells (Figure 6A–6C). We next examined the effects of inhibiting SATB2 on the expression of cadherins, and EMT transcription factors (Figure 6D). Expression of SATB2 shRNA in Pan CSCs inhibited the expression of N-cadherin, Zeb1 and Snail, as measured by qRT-PCR and/or Western blot analysis. Furthermore, SATB2 shRNA induced the expression of E-cadherin mRNA. These data suggest that inhibition of SATB2 expression in pancreatic cancer cells can modulate EMT characteristics which were demonstrated by inhibition in cell motility, migration and invasion.

We have recently demonstrated the high expression of Bcl-2 and XIAP in Pan CSCs. Our ChIP experiments have demonstrated that SATB2 can directly bind to Bcl-2 and XIAP promoters. We therefore examined whether inhibition of SATB2 can attenuate the expression of Bcl-2 and XIAP. SATB2 shRNA inhibited the expression of Bcl-2 and XIAP at mRNA and protein levels in Pan CSCs (Figure 6E).

Transcription factors such as c-Myc, Nanog and Oct-4 are required for maintaining pluripotency and self-renewal. We therefore examined the effects of SATB2 shRNA on the expression of c-Myc, Nanog and Oct-4 in Pan CSCs. As shown in Figure 6F, SATB2 shRNA inhibited the expression of c-Myc, Nanog, and Oct-4.

Since inhibition of SATB2 shRNA inhibited cell proliferation and colony formation, we next sought to examine whether inhibition of SATB2 suppresses the expression of stem cell markers in Pan CSCs, as measured by qRT-PCR and/or Western blot analysis. As shown in Figure 6G, SATB2 shRNA inhibited the expression of CD24, CD44 and CD133. All together, these data suggest that SATB2 can regulate markers of EMT, cell proliferation, pluripotency and stem cells.

### SATB2-transformed HPNE cells are tumorigenic in Balb c nude mice

**Figure 7:**
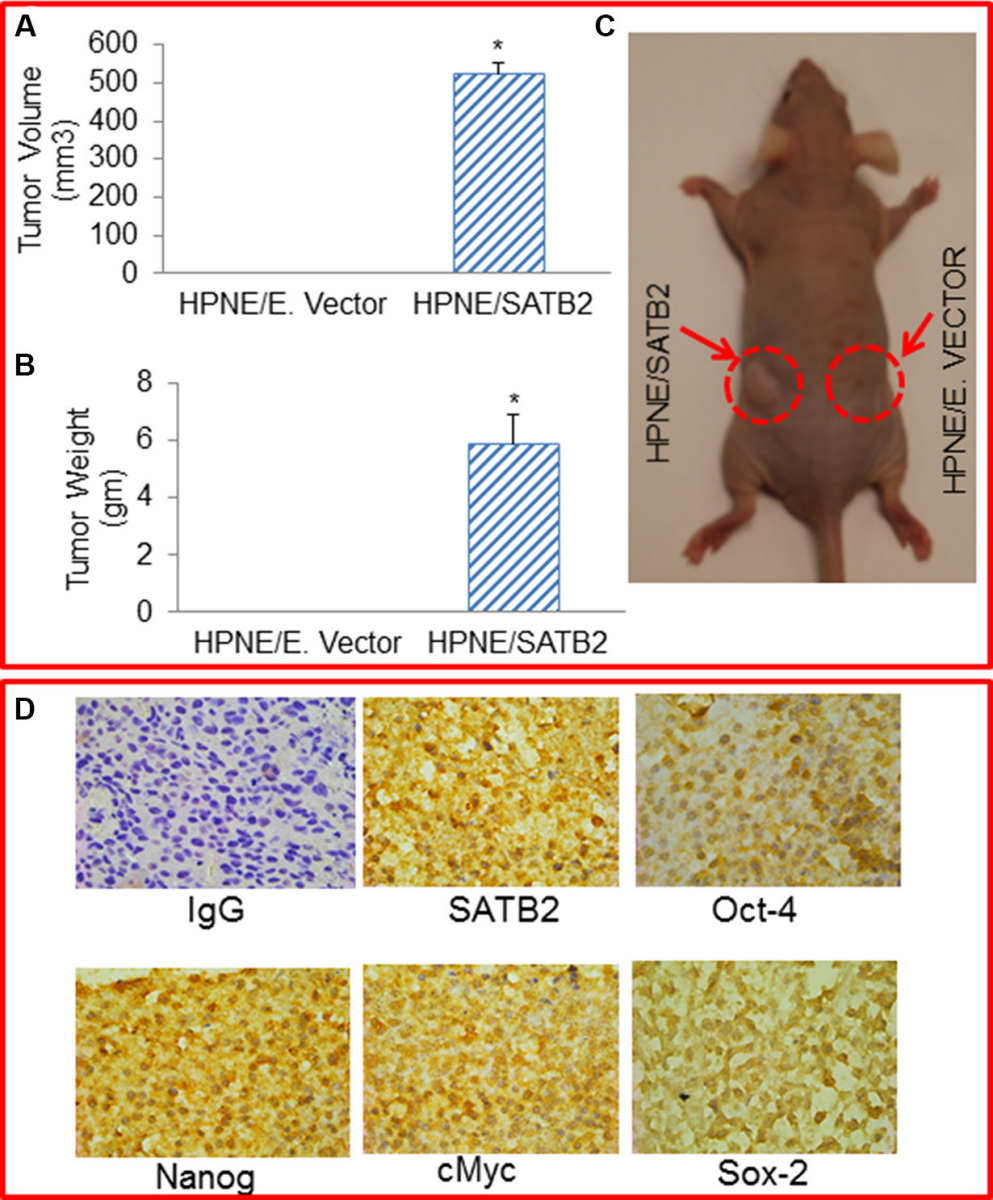
SATB2 overexpressing HPNE cells formed tumors in Balb C Nude mice (**A** and **B**) Mice (7 mice, 6 weeks old) were sc injected with HPNE/empty vector and HPNE/SATB2 cDNA cells (1 × 10^6^ cells mixed with Matrigel, 50:50 ratio, 100 μl total volume) into the right and left flanks, respectively. After 37 days, mice were sacrificed, and tumor volume and weight were measured. Data represent mean (*n* = 7) ± SD. * = significantly different from HPNE/empty vector group/control (*P* < 0.05). (**C**) A tumor-bearing mice. Left flank - HPNE/SATB2 cells formed tumor, Right flank - no tumor formation by HPNE/empty vector cells. (**D**) Immunohistochemistry of tumor tissues. Tumor tissues were harvested from HPNE/SATB2 groups as described above and immunohistochemistry was performed to measure the expression of SATB2, Oct-4, Nanog, c-Myc and Sox-2. IgG was used as a negative control.

Tumor formation is one of the properties of transformed cells. We therefore examined whether overexpression of SATB2 gene in normal HPNE cells will transform them so that they can become tumorigenic in Balb C nude mice. HPNE/SATB2 and HPNE/empty vector cells were injected into the flanks of Balb C Nude mice. SATB2 overexpressing HPNE cells (HPNE/SATB2) formed tumors (evident by tumor volume and tumor weight) in Nude mice, whereas HPNE/Empty vector cells did not form any tumor in mice (Figure 7A–7C). We next examined the expression of SATB2, Oct-4, Nanog, c-Myc and Sox-2 in tumor tissues harvested from HPNE/SATB2 groups by immunohistochemistry (Figure 7D). HPNE/SATB2 groups expressed high levels of SATB2, Oct-4, Nanog, c-Myc and Sox-2 proteins in tumors. These data suggest that SATB2 can induce malignant transformation in HPNE cells which are capable of forming tumors with high levels of Oct-4, Nanog, c-Myc and Sox-2 expression.

### SATB2 is highly expressed in human pancreatic cancer tissues

**Figure 8:**
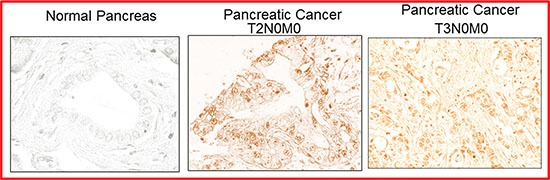
Expression of SATB2 in human pancreatic cancer tissues Human pancreatic tissue arrays containing normal and cancerous tissues (*n* = 24) were purchased from US Biomax. The expression of SATB2 protein was measured by IHC. Representative photograph of pancreatic tissues. Light Blue = nuclei, Brown/pink color = SATB2.

Since SATB2 is expressed in pancreatic cancer cell lines and Pan CSCs, it could be used as a diagnostic or predictive biomarker of pancreatic cancer. We therefore compared the expression SATB2 in human pancreatic normal and cancerous tissues (T2N0M0 and T3N0M0). Immunohistochemistry (IHC) for SATB2 on pancreatic normal and cancerous (*n* = 24) tissue microarray (TMA) was performed. The representative images for SATB2 expression in TMA are shown in Figure 8. Human pancreatic normal tissues did not express SATB2 protein. In contrast, human pancreatic cancer tissues expressed high levels of SATB2 protein. These data suggest that SATB2 protein is expressed in human pancreatic ductal adenocarcinoma and can be used as a diagnostic biomarker of pancreatic cancer.

## DISCUSSION

We have demonstrated for the first time that overexpression of SATB2 alone is capable of inducing dedifferentiation/stemness in human pancreatic normal ductal epithelial (HPNE) cells. These transformed cells gained the phenotype of cancer stem cells or progenitor cells as they express pluripotency maintaining factors (Nanog, Oct-4, Sox-2 and c-Myc). These SATB2-transformed cells formed spheroid in suspension and also gained EMT characteristics which were evident by down-regulation of E-cadherin and up-regulation N-cadherin expression, and enhanced cell motility, migration and invasion. Overall our study suggests that SATB2 gene alone can induce oncogenic transformation and play a significant role in pancreatic carcinogenesis.

There has been considerable long-standing interest in trans-differentiation as a method for the generation of desired cell types [26, 27]. The generation of iPSC by Yamanaka and colleagues through ectopic expression of four “pluripotency factors” (Oct4, Sox2, Klf4 and c-Myc) [28] has caused a resurgence of interest in cellular reprogramming. Several studies have now demonstrated that expression of lineage-specific regulatory genes can promote direct conversion or trans-differentiation from one mature differentiated cell type into a distinct differentiated cell type [29–34]. Alternatively, a “primed conversion” or “indirect lineage conversion” approach [26, 27] uses transient expression of pluripotency factors to induce a plastic developmental state permissive for respecification of desired cell fates after exposure to appropriate external cues, such as specific cell culture conditions [35, 36]. For example, neural progenitors generated by this approach can be expanded in culture and generate different neuronal and glial types after multiple passages [36, 37]. Thus, pluripotency factors can induce an epigenetically unstable state that is responsive to environmental signals and can be directed to lineage-specific progenitors and differentiated derivatives. Furthermore, combining this approach with expression of lineage-specific regulatory genes could provide additional specificity of direct conversion.

Overexpression of SATB2 in HPNE cells induces dedifferentiation/stem cell phenotypes. SATB2- overexpressing HPNE cells gained the ability to express pluripotency maintaining factors and stem cell markers, form spheroids and colonies, and undergo epithelial to mesenchymal transition *in vitro*. Our ChIP data demonstrated that SATB2 can directly bind to Bcl-2, Bsp, Nanog, XIAP, Klf4 and Hoxa2 which regulate stemness, cell growth and survival. Conversely, knockdown of SATB2 gene in pancreatic CSCs inhibited cell proliferation, spheroid and colony formation, EMT and expression of pluripotency (c-Myc, Nanog and Oct-4) and stem cell markers (CD24, CD44 and CD133). Furthermore, SATB2-overexpressing HPNE cells were able to form tumors in mice, whereas HPNE/empty vector cells did not form any tumors. Tumors isolated from HPNE/SATB2 groups expressed SATB2, Oct-4, Nanog, c-Myc and Sox-2, suggesting the maintenance of stem cell properties by these tumors.

Cancer cell invasion, dissemination and metastasis is triggered by an aberrant activation of EMT [19]. The process of EMT is mediated by the transcription factors (Zeb1, Snail and Slug) which negatively regulates the expression of E-cadherin [25]. Disseminating cancer cells must acquire specific characteristics that allow them to reestablish at different organ sites [25]. In the present study, SATB2 overexpression promoted EMT and its inhibition suppressed/reversed EMT characteristics. Specifically, SATB2 shRNA inhibited the expression of Zeb1, Snail and N-cadherin, and induced the expression of E-cadherin, a phenomena known as reversal of EMT. These data suggest that SATB2 has potential to enhance EMT and metastasis.

We have analyzed the promoters of SATB2, Nanog, Oct-4, c-Myc, Sox-2 and Klf4. We found that SATB2 promoter contains Oct-4, Nanog and c-Myc binding sites. Interestingly, SATB2 binding sites are present in Nanog, Oct-4, c-Myc, Sox2 and Klf4. These predicted models suggest that Oct-4, Myc and Nanog can regulate SATB2; and SATB2 can regulate Nanog, Oct-4, c-Myc, Sox-2 and Klf-4. Thus regulation of SATB2 can modulate the expression of pluripotency maintaining factors Nanog, Oct-4, c-Myc, Sox-2 and Klf-4. Since SATB2 binding sites are present in the promoters of Nanog, Oct-4, SOX-2 and Klf-4 (pluripotency genes), which suggest that SATB2 can act as a master regulator of pluripotency in CSCs. The biological functions of SATB2 in pancreatic cancer initiation, progression and metastasis have never been examined. Our studies will enhance our understanding of the role of SATB2 in malignant transformation, tumor growth and metastasis.

In conclusion, our data demonstrate that overexpression of SATB2 induces HPNE cell transformation, and these transformed cells possess properties of progenitor cells/CSCs. *In vivo*, SATB2-overexpressing HPNE cells formed tumors which expressed pluripotency maintaining factors. The transformed HPNE cells appear to be genotypically and phenotypically similar to progenitor cells/CSCs found in the human and mouse pancreas. We also demonstrate that SATB2 is not expressed in normal HPNE cells, but it is highly expressed in human pancreatic cancer cell lines, CSCs and primary tissues. Inhibition of SATB2 in pancreatic cancer cells suppresses cell proliferation, colony formation, cell motility, migration and invasion. SATB2 can regulate pluripotency and cell survival by modulating the expression of Bcl2, XIAP, Bsp, Klf4,c-Myc, Hoxa2 and Nanog genes. Future studies are needed to elucidate the molecular mechanisms by which SATB2 modulate pancreatic carcinogenesis, and assess its clinical significance.

## MATERIALS AND METHODS

### Cell culture conditions and reagents

AsPC-1, PANC-1, BxPC-3, Mia Paca-2, and human normal pancreatic ductal epithelial (HPNE) cells were purchased from American Type Culture Collection (ATCC), Manassas, VA. HPNE cells are immortalized cells and do not form tumors in mice. Pancreatic cancer cell lines were grown in Dulbecco's Modified Eagle's Medium with 10% Fetal Bovine Serum with antibiotics. HPNE cells were grown in well-defined cell culture medium as described [6]. Human pancreatic CSCs were characterized and grown as described elsewhere [7]. Antibodies against SATB2 and β-actin were purchased from Abcam (Cambridge, MA). Enhanced chemiluminescence (ECL) Western blot detection reagents were purchased from Amersham Life Sciences Inc. (Arlington Heights, IL). A set of GIPZ lentiviral shRNA plasmids against human SATB2 and scrambled shRNA plasmids were purchased from Open Biosystems (Lafayette, CO). SATB2 cDNA was PCR amplified from human cDNA and cloned into NheI/EcoRI digested pSicoR(Addgene). Human pancreatic tissue arrays containing normal and cancerous tissues were purchased from US Biomax (Rockville, MD).

### Lentiviral particle production and transduction

The protocol for lentivirus production and transduction have been described elsewhere [38, 39]. In brief, lentivirus was produced by triple transfection of HEK 293T cells. Packaging 293T cells were plated in 10-cm plates at a cell density of 5 **×** 10^6^ a day prior to transfection in DMEM containing 10% heat-inactivated fetal bovine serum. 293T cells were transfected with 4 μg of plasmid and 4 μg of lentiviral vector using lipid transfection (Lipofectamine-2000/Plus reagent, Invitrogen) according to the manufacturer's protocol. Viral supernatants were collected and concentrated by adding PEG-it virus precipitation solution (SBI System Biosciences) to produce virus stocks with titers of 1 **×** 10^8^ to 1 **×** 10^9^ infectious units per ml. Viral supernatant was collected for three days by ultracentrifugation and concentrated 100-fold. Titers were determined on 293T cells. Cells were transduced with lentiviral particles expressing gene of interest.

### Motility assay

We used scratch motility assay to monitor the horizontal movement of cells as described elsewhere [40].

### Transwell migration assay

Transwell migration assay was performed as described elsewhere [38].

### Transwell invasion assay

Transwell invasion assay was performed as described elsewhere [38].

### Western blot analysis

The western blot analysis was performed as we described earlier [41]. In brief, cell lysates were subjected to SDS-PAGE, and gels were blotted on nitrocellulose membrane (Amersham Biosciences, Piscataway, NJ, USA). The membranes were blocked with 5% BSA in Tris-Tween buffered saline at 37°C for 2 h and then incubated with primary antibody diluted in tris-buffered saline (1:1000 dilutions) overnight at 4°C, with gentle shaking. The membranes were then washed three times with tris-buffered saline-T (TBS-T) and incubated with secondary antibody linked to horseradish peroxidase (1:5000) for 1 h. After incubation with secondary antibody, the membranes were washed again three times with TBS-T. Finally, protein antibody complexes were detected by the addition of ECL substrate (Thermo Fisher Scientific, Rockford, IL).

### Chromatin immunoprecipitation (ChIP) assay

Pancreatic CSCs were fixed with 1% formaldehyde for 15 min (RT), quenched with 125 mM glycine for 5 min (RT), centrifuged and resuspended in RIPA Buffer containing protease inhibitors and incubated on ice (10 min). Samples were sonicated (Heat Systems-Ultrasonic device) to shear chromatin to an average length of about 1 Kb and transferred to 1.5 ml tubes, microcentrifuged for 10 min (max speed). Supernatants were collected in 1.5 mL tubes containing 1 ml of the Dilution Buffer (0.01% SDS, 1.1% Triton, 1.2 mM EDTA, 167 mM NaCl, 17 mM Tris, pH 8). 3 μg of SATB2 antibody was added to the samples, and the mixtures were incubated overnight at 4°C, followed by addition of 5 μl of protein-A and protein-G magnetic beads (Invitrogen) for 2 h. Beads were collected with a magnet (Thermo), washed 4X with 1 ml of each of four Wash Buffers (Wash Buffer 1: 0.1% SDS, 1% Triton, 2 mM EDTA, 150 mM NaCl, 20 mM Tris, pH 8; Wash Buffer 2: 0.1% SDS, 1% Triton, 2 mM EDTA, 500 mM NaCl, 20 mM Tris, pH 8; Wash Buffer 3: 0.25 M LiCl, 1% NP-40, 1% deoxycholate, 1 mM EDTA, 10 mM Tris, pH 8; Wash Buffer 4:10 mM Tris, pH 8, 1 mM EDTA). After the last wash, 50 μl of a 10% Chelex-100(Bio-Rad) resin solution was added to the beads, and samples were boiled (10 min) and microcentrifuged (1 min, max speed). After collecting supernatant, 50 μl of MQ water was added back to the beads, microcentrifuged again (1 min, max speed), and the new supernatant pooled with the previous one. 1–3 μl elutions were used for PCR reaction. The sequence of gene-specific primers are given in Supplementary Information.

### Quantitative real-time PCR

Total RNA was isolated using an RNeasy Mini Kit (Qiagen, Valencia, CA). Briefly, cDNA was synthesized using a high capacity cDNA reverse transcription kit (Applied Biosystems). Primers specific for each of the signaling molecules were designed using NCBI/Primer-BLAST and used to generate the PCR products. For the quantification of gene amplification, Real-time PCR was performed using an ABI 7300 Sequence Detection System in the presence of SYBR-Green. The sequence of gene-specific primers are given in Supplementary Information. Target sequences were amplified at 95°C for 10 min, followed by 40 cycles of 95°C for 15 s and 60°C for 1 min. HK-GAPD was used as endogenous normalization control. All assays were performed in triplicate and were calculated on the basis of ΔΔCt method. The fold change in mRNAs expression was determined according to the method of 2^−ΔΔCT^.

### Immunofluorescence and immunohistochemistry

For immunofluorescence staining, cells were grown on fibronectin-coated coverslips (Becton Dickinson, Bedford, MA). Cells were fixed with methanol, permeabilized with 1% NP-40, and blocked with 10% BSA, followed by incubating with primary antibody. After washing, cells were incubated with FITC-labeled secondary antibody. Finally, coverslips were washed and mounted using Vectashield (Vector Laboratories, Burlington, CA). Stained slides were examined under a fluorescence microscope. Cells without primary antibody, or with Isotype-specific control IgG, were used as negative controls. Human pancreatic normal and cancer tissue arrays were purchased from US Biomax, Inc. (Rockville, MD). Immunohistochemistry of human normal and tumor tissues was performed as described elsewhere [38].

### *In vivo* xenograft experiment

HPNE cells were stably transduced with either empty vector (HPNE/empty vector) or SATB2 cDNA (HPNE/SATB2 cDNA). Balb C Nude mice were purchased from the Jackson's Laboratory (Bar Harbor, Maine), and they (7 mice, 6 weeks old) were subcutaneously injected with HPNE/empty vector and HPNE/SATB2 cells (1 × 10^6^ cells mixed with Matrigel, 50:50 ratio, 100 μl total volume) into right and left flanks, respectively. After 37 days, mice were sacrificed. Tumor volume and weight were recorded. All experiments on mice in this study were performed with the approval of and in accordance with, the Institutional Animal Care and Use Committee's protocol.

### Statistical analysis

The mean and SD were calculated for each experimental group with replicates. Differences between groups were analyzed by ANOVA, followed by Bonferoni's multiple comparison tests using PRISM statistical analysis software (GrafPad Software, Inc., San Diego, CA). Significant differences among groups were calculated at *P* < 0.05.

## SUPPLEMENTARY MATERIALS


