# Supplementary material for: Role of SATB2 in human pancreatic cancer: Implications in transformation and a promising biomarker
Source: Oncotarget. 2016 Jul 27;7(36):57783–97. doi: 10.18632/oncotarget.10860 (PMC5295389; doi:10.18632/oncotarget.10860)
Supplement: Supplementary file 1 [file oncotarget-07-57783-s001.pdf]

Role of SATB2 in human pancreatic cancer: Implications in transformation and a promising biomarker

Supplementary Materials

MATERIALS AND METHODS

Lentiviral particle production and transduction

Lentivirus was produced by triple transfection of HEK 293T cells. Packaging 293T cells were plated in 10-cm plates at a cell density of 5 × 10^6 a day prior to transfection in DMEM containing 10% heat-inactivated fetal bovine serum. 293T cells were transfected with 4 µg of plasmid and 4 µg of lentiviral vector using lipid transfection (Lipofectamine-2000/Plus reagent, Invitrogen) according to the manufacturer's protocol. Viral supernatants were collected and concentrated by adding PEG-it virus precipitation solution (SBI System Biosciences) to produce virus stocks with titers of 1 × 10^8 to 1 × 10^9 infectious units per ml. Viral supernatant was collected for three days by ultracentrifugation and concentrated 100-fold. Titers were determined on 293T cells. Pancreatic cancer cells were transduced with lentiviral particles expressing gene of interest.

Motility assay

A monolayer of cells was established and then a scratch was made through the monolayer which gave rise to an *in vitro* wound. Wells were washed twice with PBS, and replaced with fresh culture medium. Movement of cells to the scratch area as single cells from the confluent sides was monitored. The width of the scratch gap is viewed under the microscope in four separate areas each day until the gap is completely filled in the untreated control wells. Three replicate wells from a 6-well plate were used for each experimental condition.

Transwell migration assay

In brief, 1 × 10^5 pancreatic cancer cells were plated in the top chamber onto the noncoated membrane (24-well insert; pore size, 8 µm; Corning Costar) and allowed to migrate toward serum-containing medium in the lower chamber. Cells were fixed after 24 hours of incubation with methanol and stained with 0.1% crystal violet (2 mg/ml, Sigma-Aldrich). The number of cells invading through the membrane was counted under a light microscope.

Transwell invasion assay

In brief, 1 × 10^5 cells were plated in the top chamber onto the Matrigel coated Membrane (24-well insert; pore size, 8 µm; Corning Costar). Each well was coated freshly with Matrigel (60 µg; BD Bioscience) before the invasion assay. Pancreatic cancer cells were plated in medium without serum or growth factors, and medium supplemented with serum was used as a chemoattractant in the lower chamber. The cells were incubated for 48 hours and cells that did not invade through the pores were removed by a cotton swab. Cells on the lower surface of the membrane were fixed with methanol and stained with crystal violet. The number of cells invading through the membrane was counted under a light microscope (40×, three random fields per well).

Western blot analysis

Cell lysates were subjected to SDS-PAGE, and gels were blotted on nitrocellulose membrane (Amersham Biosciences, Piscataway, NJ, USA). The membranes were blocked with 5% BSA in Tris-Tween buffered saline at 37°C for 2 h and then incubated with primary antibody diluted in tris-buffered saline (1:1000 dilutions) overnight at 4°C, with gentle shaking. The membranes were then washed three times with tris-buffered saline-T (TBS-T) and incubated with secondary antibody linked to horseradish peroxidase (1:5000) for 1 h. After incubation with secondary antibody, the membranes were washed again three times with TBS-T. Finally, protein antibody complexes were detected by the addition of ECL substrate (Thermo Fisher Scientific, Rockford, IL).

Chromatin immunoprecipitation (ChIP) assay

Pancreatic CSCs were fixed with 1% formaldehyde for 15 min (RT), quenched with 125 mM glycine for 5 min (RT), centrifuged and resuspended in RIPA Buffer containing protease inhibitors and incubated on ice (10 min). Samples were sonicated (Heat Systems-Ultrasonic device) to shear chromatin to an average length of about 1 Kb and transferred to 1.5 ml tubes, microcentrifuged for 10 min (max speed). Supernatants were collected in 1.5 mL tubes containing 1 ml of the

Dilution Buffer (0.01% SDS, 1.1% Triton, 1.2 mM EDTA, 167 mM NaCl, 17 mM Tris, pH 8). 3 µg of SATB2 antibody was added to the samples, and the mixtures were incubated overnight at 4°C, followed by addition of 5 µl of protein-A and protein-G magnetic beads (Invitrogen) for 2 h. Beads were collected with a magnet (Thermo), washed 4X with 1 ml of each of four Wash Buffers (Wash Buffer 1: 0.1% SDS, 1% Triton, 2 mM EDTA, 150 mM NaCl, 20 mM Tris, pH 8; Wash Buffer 2: 0.1% SDS, 1% Triton, 2 mM EDTA, 500 mM NaCl, 20 mM Tris, pH 8; Wash Buffer 3: 0.25 M LiCl, 1% NP-40, 1% deoxycholate, 1 mM EDTA, 10 mM Tris, pH 8; Wash Buffer 4:10 mM Tris, pH 8, 1 mM EDTA). After the last wash, 50 ul of a 10% Chelex-100 (Bio-Rad) resin solution was added to the beads, samples were boiled (10 min) in a heat block, microcentrifuged (1 min, max speed), supernatants followed by the addition of 50 µl of MQ water back to the beads, microcentrifuged again (1 min, max speed), and the new supernatant pooled with the previous one. 1–3 µl elutions were used for PCR reaction.

NANOG PROMOTER FOR CHIP –F, ACCTAGTCTGGGTTACTCTGC

NANOG PROMOTER FOR CHIP –R, TCAAGAAATTGGGATAAAGTG

Bcl2 promoterChIP-F, TTTCAGCATCACAGAGGAAG

Bcl2 promoterChIP-R, CAATCACGCGGAACACTTGATT

Hoxa2-ChIP-F, CCGTCCGAAAGCATTAAGTT

Hoxa2-ChIP-R, ATGATCGCCTACAAGCGGTTCTC

BSP-Chip-F, AATGTAAAATTATATCTAATGACAGC

BSP-ChIP-R, AAACGTGATTCTAGAGCCAAGAGG

oct4-ChIP-F, ATGACCACTGCGCCCGGACTGC

oct4-ChIP-R, ACTTGGATCTCTTCCAAGTGC

Klf4-Chip-F, ACCGGACCTACTTACTCGCC

Klf4-Chip-R, TCGGCAGCCCGAAGCAGCTGG

Myc-Chip-F, AATTAATGCCTGGAAGGCAGCC

Myc-ChIP-R, AGTCAGCAGAGACCCTTGTG

Xiap-Chip-F,TCCAAGAGAGATGCACTAGGGTC

Xiap-Chip-R, TTATGGCAAGATCTATGTGGAACTC

Quantitative real-time PCR

Total RNA was isolated using an RNeasy Mini Kit (Qiagen, Valencia, CA). Briefly, cDNA was synthesized using a high capacity cDNA reverse transcription kit (Applied Biosystems). Primers specific for each of the signaling molecules were designed using NCBI/Primer-BLAST and used to generate the PCR products. For the quantification of gene amplification, Real-time PCR was performed using an ABI 7300 Sequence Detection System in the presence of SYBR- Green. The following gene-specific primers were used:

Snail (5′-ACC CCA CAT CCT TCT CAC TG -3′, 5′- TAC AAA AAC CCA CGC AGA CA -3′)

Slug (5′-ACA CAC ACA CAC CCA CAG AG -3′, 5′- AAA TGA TTT GGC AGC AAT GT -3′)

Zeb1 (5′-GCA CAA CCA AGT GCA GAA GA-3′, 5′-CAT TTG CAG ATT GAG GCT GA-3′)

E-cadherin (5’-TGC TCT TGC TGT TTC TTC GG-3′, 5′-TGC CCC ATT CGT TCA AGT AG-3′)

N-cadherin (5′-TGG ATG GAC CTT ATG TTG CT-3′, 5′- AAC ACC TGT CTT GGG ATC AA-3′)

Nanog (5′-ACC TAC CTA CCC CAG CCT TT -3′, 5′- CAT GCA GGA CTG CAG AGA TT -3′)

Sox-2 (5′- AAC CCC AAG ATG CAC AAC TC -3′, 5′- GCT TAG CCT CGT CGA TGA AC -3′)

cMyc (5′- CGA CGA GAC CTT CAT CAA AA -3′, 5′- TGC TGT CGT TGA GAG GGT AG -3′)

Oct4 (5′- GGA CCA GTG TCC TTT CCT CT -3′, 5′- CCA GGT TTT CTT TCC CTA GC -3′)

HK-GAPD (5′-GAG TCA ACG GAT TTG GTC GT-3′, 5′-TTG ATT TTG GAG GGA TCT CG-3′).

Target sequences were amplified at 95°C for 10 min, followed by 40 cycles of 95°C for 15 s and 60°C for 1 min. HK-GAPD was used as endogenous normalization control. All assays were performed in triplicate and were calculated on the basis of $\Delta\Delta Ct$ method. The n-fold change in mRNAs expression was determined according to the method of $2^{-\Delta\Delta CT}$.
